# Analysis of the rs3807135, rs3757385 and rs3778754 Variants of the *IRF5* Gene and mRNA Expression in Patients with Melanoma Cancer from Western Mexico

**DOI:** 10.3390/genes17030254

**Published:** 2026-02-24

**Authors:** Claudia A. Tapia-Leyva, Fernando Valdez-Salazar, Luis A. Jiménez-Del Río, Jorge R. Padilla-Gutiérrez, José F. Muñoz-Valle, Emmanuel Valdés-Alvarado

**Affiliations:** 1 Instituto de Investigación en Ciencias Biomédicas (IICB), Centro Universitario de Ciencias de la Salud, Universidad de Guadalajara, Guadalajara 44340, Mexico; claudia.tapia9876@alumnos.udg.mx (C.A.T.-L.); fernando.valdez9882@alumnos.udg.mx (F.V.-S.); luis.jimenez4751@alumnos.udg.mx (L.A.J.-D.R.); ramon.padilla@academicos.udg.mx (J.R.P.-G.); drjosefranciscomv@cucs.udg.mx (J.F.M.-V.); 2 Doctorado en Genética Humana, Departamento de Biología Molecular y Genómica, Universidad de Guadalajara, Guadalajara 44340, Mexico

**Keywords:** melanoma, *IRF5*, variants, expresion, México population

## Abstract

Objective: To analyze the association between the *IRF5* gene variants rs3807135, rs3757385, and rs3778754 and mRNA expression levels in patients from western Mexico diagnosed with melanoma. Methods: An analytical cross-sectional study was conducted including 374 individuals (153 patients with newly diagnosed melanoma and no previous treatment, and 221 controls). The melanoma group was matched to the reference group. Genotyping of the rs3807135 (T>C), rs3757385 (T>G), and rs3778754 (C>G) variants was performed using the allelic discrimination method with TaqMan^®^ probes. Relative mRNA expression was quantified by qPCR using the 2^–ΔΔCT^ method, comparing *IRF5* expression levels with those of the housekeeping gene *GAPDH*. Statistical analyses were performed in R, and allelic and genotypic frequencies were compared between patients and controls using the Chi-square test. Results: No statistically significant associations were identified between *IRF5* SNVs rs3807135, rs3757385, and rs3778754 and melanoma risk. The haplotypic pattern comprised TTC, CGG, and CGC, with CGG showing a non-significant protective tendency. The mean relative expression of *IRF5* was lower in melanoma patients compared with controls (≈0.39 vs. 1.0; Δ = 0.61), although this difference did not reach statistical significance (U = 1725; *p* = 0.841). These findings suggest a modest modulatory effect of *IRF5* at the haplotypic level, likely driven by combined variant effects. Conclusions: In conclusion, the present study did not identify statistically significant associations between the *IRF5* single-nucleotide variants rs3807135, rs3757385, and rs3778754 and melanoma risk in the analyzed population from western Mexico. Likewise, no significant differences in allele or genotype distributions were observed between melanoma patients and control individuals. These findings suggest that the evaluated *IRF5* genetic variants do not constitute major susceptibility factors for melanoma in this cohort.

## 1. Introduction

The skin is the largest organ of the human body, representing up to 15% of total body weight in adults and performing specialized functions such as thermoregulation, sensory perception, water storage, vitamin D synthesis, expression, and communication. Its main role, however, is protection, acting as a physical barrier between the body and its external environment. It also plays a key role in the immune system by defending against pathogens and, notably, by protecting against solar exposure and the damage caused by ultraviolet (UV) radiation [1].

The skin is organized into three layers (hypodermis, dermis, and epidermis), with the latter composed mainly of keratinocytes, melanocytes, Langerhans cells, and Merkel cells [2]. Due to continuous exposure to diverse damaging agents, these cells can acquire alterations that may confer neoplastic potential. Cutaneous cancer (CC) is a multifactorial disease influenced primarily by environmental factors. Based on the affected cell type, it is divided into two main categories: melanoma and non-melanoma [3].

Melanoma arises from uncontrolled proliferation of melanocytes and, although less common, is clinically significant because of its high aggressiveness and propensity for lymphatic or hematogenous metastasis. Excessive exposure to UV radiation (whether from sunlight or tanning beds) is a major risk factor for melanoma development [4]. Genetic predisposition also plays a role, with mutations in genes such as *CDKN2A* (which encodes the tumor-suppressor proteins p14 and p16) and, less frequently, *IL10*, *PDCD1*, and *CDK4* (a kinase crucial for cell-cycle regulation) increasing susceptibility [5,6,7]. According to [8], 331,722 new melanoma cases were reported worldwide, ranking 17th among all cancer types, with 58,667 related deaths, ranking 22nd as a cause of cancer mortality [8].

Melanoma incidence continues to rise annually, largely due to increased UV exposure. Although environmental factors are key, the contribution of genetic and immunological components to melanoma development and progression is becoming increasingly recognized [9].

In this context, the *IRF5* gene stands out as it encodes a member of the interferon regulatory factor family, a transcription factor critical for innate immune regulation through the induction of type I interferons (IFN-α and IFN-β). *IRF5* is constitutively expressed in lymphoid tissues such as the spleen and in immune cells, particularly monocytes, macrophages, B cells, and NK cells, though to a lesser extent in T cells. Its functional relevance lies in macrophage polarization toward the M1 proinflammatory phenotype, as well as the induction of proinflammatory cytokines (IL-6, IL-12, IL-23, and TNF-α) and chemokines involved in T-cell recruitment [10,11,12,13,14,15].

IRF5 protein promotes apoptosis following genomic damage by regulating pro-apoptotic genes such as *BAK1*, *BAX*, *CASP8*, and *DAPK2* [15,16]. Experimental models have linked IRF5 downregulation to enhanced proliferation, migration, invasion, metastatic burden, and reduced survival [17,18]. The *IRF5* gene, located on chromosome 7q32.1 (positions 128,937,457–128,950,038) [19], contains nine exons. Its N-terminal region comprises a DNA-binding domain with five characteristic tryptophan residues typical of the IRF family, while the C-terminal region harbors a regulatory domain (IAD) responsible for protein–protein interactions with other IRF members or transcription factors [13,20,21].

*IRF5* is highly polymorphic and undergoes alternative splicing, generating multiple functional isoforms (V1–V11) that confer structural and functional diversity across cell types and physiological contexts [15,20]. Single-nucleotide variants (SNVs) in *IRF5* have been associated with various autoimmune diseases [22], including systemic lupus erythematosus [23], rheumatoid arthritis [24], multiple sclerosis [25], systemic sclerosis [26,27], and Sjögren’s syndrome [28], and more recently with cancer. Certain variants (rs3807135, rs3757385, rs3778754) have been shown to affect *IRF5* expression, mRNA stability, and inflammatory pathway activation.

In skin cancer contexts, *IRF5* expression correlates with local immune responses and tumor biology, where *IRF5* loss or mutation may impair antigen presentation and reduce the effectiveness of immunotherapy. For instance, absence of the A allele in rs10954213 (a 3′UTR variant) has been associated with poorer responses to adoptive T-cell therapy in metastatic melanoma [29,30]. Accordingly, this study aims to investigate the association between specific *IRF5* variants (rs3807135, rs3757385, rs3778754) and mRNA expression levels with clinical features in melanoma patients from western México, to better understand the molecular mechanisms underlying these pathologies. Genotyping was performed by real-time PCR and gene expression quantified by qPCR using TaqMan^®^ probes. Statistical analyses were applied to determine associations with clinically relevant variables.

## 2. Materials and Methods

### 2.1. Study Design

The present study aimed to analyze whether the *IRF5* gene variants rs3807135, rs3757385, and rs3778754 and their expression levels were associated with melanoma in a population from western Mexico, as well as with the clinical characteristics of the disease. Given the immunological surveillance functions of *IRF5*, identifying these associations could provide insight into melanoma biology. An analytical cross-sectional design was employed, encompassing both genotyping and expression analyses using peripheral blood samples obtained from individuals in the study cohort.

#### Study Population

The study included a total of 374 individuals, comprising 153 patients diagnosed with melanoma who attended the Instituto Dermatológico de Jalisco “Dr. José Barba Rubio” and the Hospital Civil “Fray Antonio Alcalde”. All melanoma patients were newly diagnosed and had not received any prior treatment at the time of recruitment.

The reference group consisted of 221 healthy individuals recruited from the same geographic region (western Mexico) as the patient groups. These subjects were selected based on the absence of personal history of melanoma, non-melanoma skin cancer, or any other type of cancer. Additional exclusion criteria included the presence of immunosuppressive conditions or a history of recent blood transfusions. Every individual from the reference group provided written informed consent.

Melanoma patients and individuals from the reference group were frequency-matched by age and sex to ensure comparability between groups. Peripheral blood samples were collected and processed following the same standardized protocol in both groups.

### 2.2. Inclusion and Exclusion Criteria

#### 2.2.1. Inclusion Criteria

Patients who were histologically and dermatoscopically diagnosed with cutaneous melanoma, were aged 18 years or older, and provided written informed consent were included. All patients and controls were born in western Mexico (Jalisco, Nayarit, Michoacán, and/or Colima) and had at least two generations of ancestry (parents and grandparents) from the same region. The reference group consisted of apparently healthy individuals with no personal history or diagnosis of melanoma.

#### 2.2.2. Exclusion Criteria

The exclusion criteria were individuals with a history of other types of cancer, organ transplantation, or immunosuppressive conditions, and participants who received blood transfusions within three months prior to sample collection.

#### 2.2.3. Sample Size

To calculate the minimum required sample size for variant analysis, OpenEpi version 3.017 was used, applying Fleiss’ method [31], which compares proportions between two independent groups. The resulting minimum was 68 individuals per group. The calculation was based on the lowest minor allele frequency (MAF) among the variants, corresponding to rs3778754 (0.438), assuming a 95% confidence level (1 − α = 0.95), odds ratio (OR) = 2.0, and statistical power (1 − β) = 80%. A minimum of 67 individuals (133 alleles) was required per group; thus, the total sample size exceeded the threshold, ensuring robust statistical power for *IRF5* analysis.

For the gene expression analysis, the minimum sample size was determined using the formula for comparing means between two independent groups. This resulted in a minimum of 30 samples per group. In this study, *IRF5* expression was measured in 75 melanoma samples and 45 control samples, exceeding the minimum threshold required to achieve adequate statistical power.

### 2.3. Molecular Analysis

#### 2.3.1. DNA Extraction and Quantification

Genomic DNA was isolated from total leukocytes following a modified Miller protocol [32]. A 15 mL sample of peripheral blood was collected from each participant in EDTA-coated BD Vacutainer^®^ (NJ, USA) tubes for both DNA and RNA extraction. Written informed consent was obtained, and a structured clinical questionnaire was completed for each participant. DNA and RNA concentrations and purity were determined spectrophotometrically using a NanoDrop Lite instrument (Waltham, MA, USA). All samples were stored at −20 °C until further processing.

#### 2.3.2. Genotyping

Genotyping of the *IRF5* variants rs3807135 (T>C), rs3757385 (T>G), and rs3778754 (C>G) was performed using allelic discrimination assays with TaqMan^®^ probes labeled with VIC^®^ and FAM™ fluorophores, run on a LightCycler 96^®^ real-time PCR system.

#### 2.3.3. mRNA Expression

Total RNA was extracted using the modified Chomczynski and Sacchi [33] method with TRIzol^®^ Reagent. Relative mRNA expression levels were determined by quantitative PCR (qPCR) using TaqMan^®^ (Foster City, CA, USA) probes on a LightCycler^®^ 96 real-time PCR platform (Mannheim, Germany). Complementary DNA (cDNA), previously synthesized and validated, was used as the template, and expression values were calculated using the 2^−ΔΔCt^ method, with *IRF5* expression (FAM fluorophore) normalized to the reference gene *GAPDH* (VIC fluorophore) to estimate relative transcriptional variation.

#### 2.3.4. Statistical Analysis

All statistical analyses were performed using R software (2025.09.1+401). Clinical characteristics of melanoma patients were described using absolute and relative frequencies. Allelic and genotypic frequencies between melanoma and control groups were compared using the Chi-square test (χ^2^). Hardy–Weinberg equilibrium (HWE) was assessed in the control group. Non-parametric tests were applied when distributional assumptions were not met.

#### 2.3.5. Ethical Considerations

This project constitutes an extension and follow-up analysis of a previously approved research protocol by the Research Ethics Committee, with the registration number CI-01222. That approved protocol included the collection, processing, and storage of biological samples (blood) from patients with melanoma and cancer-free individuals serving as controls, all of whom provided written informed consent.

Samples were collected in strict accordance with the Declaration of Helsinki, adopted by the 18th World Medical Assembly in Helsinki, Finland, in June 1964 and revised by the 60th General Assembly of the World Medical Association in 2013, in Fortaleza, Brazil, as well as with the Regulation of the General Health Law on Health Research [34]. According to Article 17 of the Ethical Aspects of Research in Human Subjects of the Regulation of the General Health Law on Health Research, this study is classified as minimal risk, as it involves procedures commonly performed during routine diagnostic or therapeutic physical examinations.

For this study, blood samples were obtained from patients with skin cancer and from individuals in the control group. Written informed consent was obtained from both patients and controls, and a detailed yet comprehensible explanation of the procedures carried out for the development of this research project was provided.

## 3. Results

### 3.1. Sociodemographic Characteristics

Table 1 presents the sociodemographic characteristics for a total of 374 samples analyzed, comprising 153 melanoma patients and 221 reference group individuals. The median age was 62.5 years for melanoma patients and 66 years for controls, with no statistically significant difference between groups (*p* = 0.08). Sex distribution was similar in both groups, with females representing approximately 60% and males 40%.

Regarding skin phototype, type III was the most common among controls, followed by type IV, with minimal representation of types I, II, and V. In contrast, melanoma patients exhibited a more heterogeneous distribution, with a higher frequency of phototypes IV, II, and III, respectively.

Table 2 summarizes the clinical characteristics of melanoma patients stratified by sex. No significant differences were observed between males and females for histological subtype (*p* = 0.57), Breslow thickness (*p* = 0.54), or Clark level (*p* = 0.82). However, anatomical location differed significantly between sexes (*p* = 0.05), with women showing a higher proportion of lesions on lower limbs (37.2%) compared to men (20%), and also more cases on upper limbs (22.1% vs. 16.7%). The superficial spreading subtype was the most common overall (40%).

Alleles and genotypes of the *IRF5* gene in patients with melanoma and the reference group.

Table 3 shows the genotyping results for the *IRF5* variants. In the case of the rs3807135 variant (T>C), the Hardy–Weinberg equilibrium (HWE) was confirmed in the reference group (*p* = 0.56). The variant allele (C) was the most frequent in both groups, with frequencies of 53% in the patient group and 58% in the reference group. The heterozygous genotype was the most prevalent in both groups, accounting for 47% of patients and 52% of controls. The odds ratio (OR) analysis indicated a non-significant association between the variant allele and melanoma risk (OR = 1.255; 95% CI: 0.934–1.686; *p* = 0.131).

For the rs3757385 (T>G) variant, the variant allele (G) was the most frequent in both groups, with frequencies of 53% in melanoma patients and 57% in the reference group. The heterozygous genotype was the most prevalent in both groups (47% in patients and 51% in controls), followed by the homozygous variant genotype, which was observed in 29% of patients and 32% of reference individuals. Odds ratio (OR) analysis indicated a non-significant association between the G allele and melanoma risk (OR = 1.22; 95% CI: 0.908–1.639; *p* = 0.185). Hardy–Weinberg equilibrium was confirmed in the reference group (*p* = 0.687).

For the rs3778754 (C>G) variant, the wild-type allele (C) was the most frequent in both groups, with frequencies of 56% in melanoma patients and 51% in the reference group, with no statistically significant difference between groups. The variant allele (G) was less frequent in patients than in the reference group. Odds ratio (OR) analysis showed a non-significant association with melanoma risk (OR = 0.811; 95% CI: 0.604–1.088; *p* = 0.163). Regarding genotypes, the heterozygous C/G genotype was the most prevalent in both groups, accounting for 48% of patients and 49% of reference individuals. Hardy–Weinberg equilibrium was confirmed in the reference group (*p* = 0.998).

### 3.2. Linkage Disequilibrium

**Figure 1:**
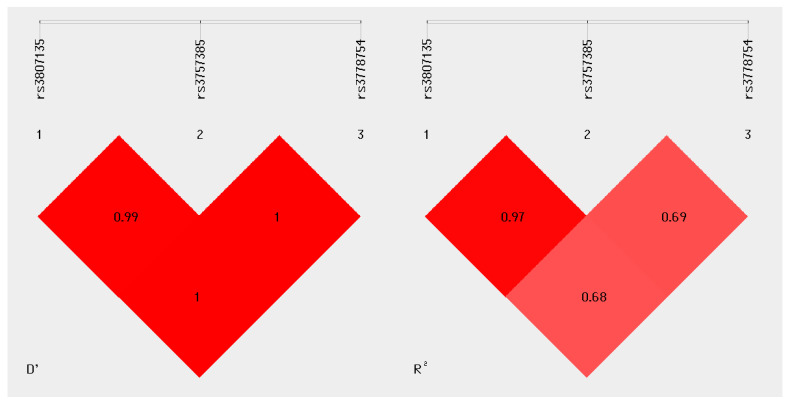
Linkage disequilibrium (LD) map of the *IRF5* gene for SNVs rs3807135, rs3757385, and rs3778754 in the analyzed cohort. Values within the cells indicate the correlation coefficient (R^2^) between SNV pairs, showing a strong association between rs3807135 and rs3757385 (R^2^ = 0.97) and complete linkage (D′ = 1.0) among the three variants. Darker shades represent higher correlation, illustrating the presence of a compact haplotype block within the *IRF5* locus.

Linkage disequilibrium (LD) analysis revealed a highly correlated block among the three variants (Figure 1). The LD value D′= 0.99 between rs3807135 and rs3757385 indicated co-segregation within the studied population. LD between rs3757385–rs3778754 and rs3807135–rs3778754 yielded D′= 1.0, indicating complete linkage. These findings indicated the absence of recombination events among the three loci. The correlation coefficient confirmed an almost perfect association between rs3807135 and rs3757385 (R^2^ = 0.97), whereas rs3778754 showed moderate correlations with the other two variants (R^2^ = 0.68–0.69), consistent with a compact haplotype block and partial allelic independence for rs3778754.

### 3.3. Haplotype Analysis

Table 4 shows the haplotype frequencies for the three variants. Haplotypes with a frequency <0.03 were excluded to avoid imprecise estimates. Three major haplotypes (TTC, CGG, and CGC) were identified, consistent with a compact haplotype structure (D′ = 1.0). None of the haplotypes showed a statistically significant association with melanoma.

### 3.4. Relative Expression of IRF5

**Figure 2:**
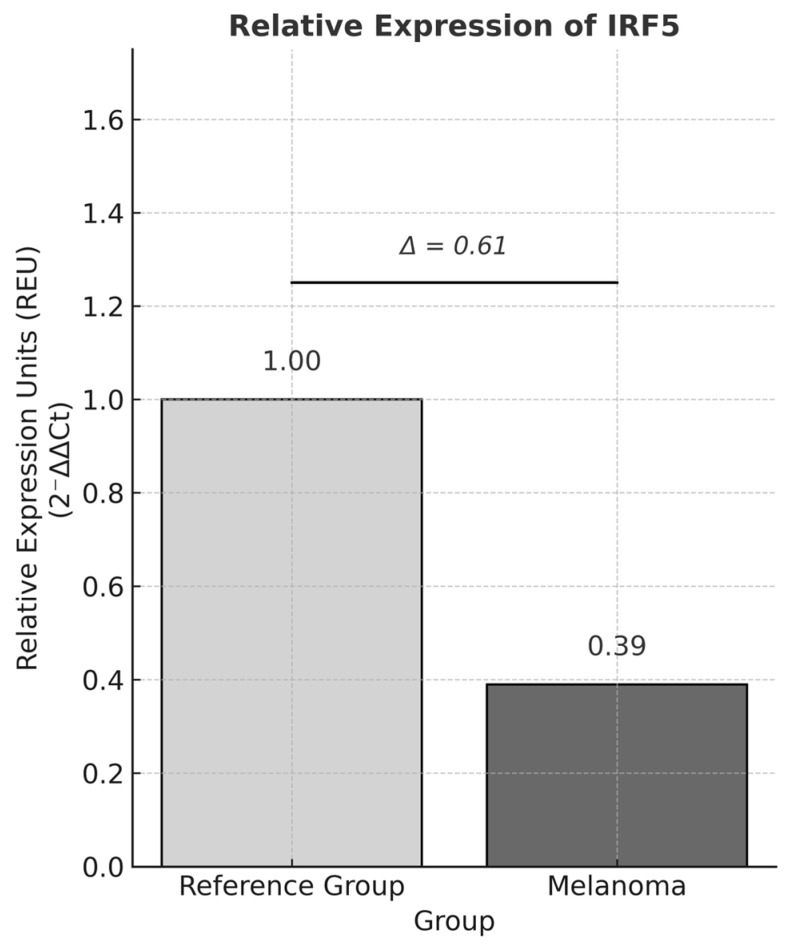
Relative expression of *IRF5* in melanoma and control samples. Bars represent mean relative expression normalized to *GAPDH* and calibrated to the control (set as 1.0) using the 2^−ΔΔCt^ method). *IRF5* expression was lower in melanoma samples (0.39-fold compared with control).

*IRF5* expression was quantified in both melanoma and control groups by qPCR using TaqMan^®^ probes. The mean relative expression in melanoma samples was 0.39 compared with 1.0 in controls, corresponding to a 61% decrease in *IRF5* expression in melanoma. These results were corroborated using Pfaffl’s method, yielding consistent relative expression values (*GAPDH* = 1.04; *IRF5* = 0.38) (Figure 2).

### 3.5. Analysis of Clinicopathological Features

To identify associations between the clinical characteristics of melanoma patients, comparative analyses were performed using contingency tables and tests of independence (Chi-square or Fisher’s exact test, as appropriate). All possible combinations were analyzed. Statistically significant associations were observed between histological subtype and Breslow thickness (Table 5), as well as between histological subtype and anatomical location (Table 6) (*p* < 0.01).

As shown in Table 5, most superficial spreading lesions were associated with a Breslow thickness ranging from <1 mm to 2 mm, whereas most nodular lesions were observed in thickness categories ranging from 2.1 mm to >4 mm. Acral lentiginous and lentigo maligna subtypes showed a more heterogeneous distribution across Breslow thickness categories. Overall, a significant association between histological subtype and tumor thickness was observed.

The comparison in Table 6 shows the distribution of melanoma subtypes according to anatomical location. Superficial spreading and nodular lesions exhibited a heterogeneous distribution across anatomical sites. In contrast, acral lentiginous lesions were predominantly observed on the lower extremities. Lentigo malign lesions were most frequently located on the head and neck. These results support how histological subtypes relate to the anatomical location of the tumor and the depth of the lesion.

It is important to note that when comparing all possible combinations of variables, no statistically significant associations were identified between *IRF5* genetic variants or *IRF5* mRNA expression levels and melanoma histopathological subtypes.

## 4. Discussion

The results of this study provide evidence supporting a potential involvement of the *IRF5* gene in the pathophysiology of melanoma, consistent with its recognized role as a key regulator of innate immunity and proinflammatory cytokine signaling. Previous research has characterized IRF5 as a critical transcription factor in type I interferon (IFN) signaling, displaying tissue-specific effects [20,35]. Recent reviews have emphasized that *IRF5* dysregulation is associated with autoimmune diseases [36]. Because of its activity in apoptosis induction, macrophage polarization and function, lymphocyte activation, and dendritic cell differentiation, IRF5 displays features that overlap with several hallmarks of cancer, suggesting its ability to modulate tumor progression and the immune microenvironment [37]. Roberts et al., 2024 [17] demonstrated that *IRF5* overactivation can induce an antitumor proinflammatory response, whereas its epigenetic or post-transcriptional repression promotes an immunosuppressive phenotype and facilitates metastatic progression. Similarly, Du et al. [38] showed that *IRF5* inactivation in gastric cancer cells was associated with reduced pulmonary metastasis in murine models.

In metastatic melanoma, Uccellini et al. [29] reported that the *IRF5* rs10954213 variant predicted the response to tumor-infiltrating lymphocyte (TIL) adoptive therapy, highlighting a possible role of *IRF5* in both metastasis and immunotherapy efficacy. In the present study, lower *IRF5* expression levels were observed in melanoma samples compared with controls; however, the difference in ΔCt values did not reach statistical significance, suggesting either biological heterogeneity within the cohort or limited statistical power to detect modest expression differences.

In this context, the reduced *IRF5* expression observed in melanoma samples may reflect a partial impairment of the IRF5–IFN axis, consistent with a less effective antitumor immune response within the tumor microenvironment. Although no statistically significant associations were identified between the *IRF5* variants rs3807135, rs3757385, and rs3778754 and melanoma risk. These findings are in line with previous evidence indicating that functional variation in *IRF5* can influence antitumor immune responses in a context-dependent manner [16,18].

Recent studies have also identified IFN/IRF signaling pathways, including homologous transcription factors such as IRF9, as regulators of immune checkpoint expression and determinants of sensitivity to immune checkpoint blockade. These findings support the concept that interindividual variability in IFN signaling may influence both tumor behavior and therapeutic response [39].

Regarding haplotype and linkage disequilibrium analyses, the combination of high D′ values (0.99–1.0) and moderate-to-high R^2^ values (0.68–0.97) suggests that rs3807135, rs3757385, and rs3778754 form a compact haplotypic block within the *IRF5* locus, with partial internal correlations. This supports the notion that haplotypic analysis may provide more representative information about *IRF5* genetic variability than single-SNV assessments. The CGG haplotype, which exhibited a non-significant protective trend (OR < 1), could warrant further evaluation in larger, independent cohorts.

Overall, while *IRF5* is not consistently identified among the most differentially expressed genes in melanoma datasets, its higher expression has been associated with a favorable prognosis, whereas lower expression correlates with immune evasion and progression. Thus, the reduced *IRF5* expression observed in this cohort could still have biological relevance, even in the absence of statistical significance.

The present findings suggest that *IRF5* acts primarily as an immune modulator rather than a direct genetic risk factor for melanoma. Nevertheless, the borderline associations detected for rs3807135 and rs3757385 justify replication studies with larger sample sizes, global haplotype analyses, and multivariate modeling. Further multi-omics integration (e.g., eQTL, ATAC-seq, and ChIP-seq analyses for *IRF5* and STAT pathways) could yield valuable mechanistic insights. As an immediate next step, functional validation is warranted to elucidate whether *IRF5* could serve as a prognostic biomarker or predictive marker for immunotherapy response, as suggested by the emerging literature.

## 5. Conclusions

In conclusion, the present study did not identify statistically significant associations between the *IRF5* single-nucleotide variants rs3807135, rs3757385, and rs3778754 and melanoma risk in the analyzed population from western Mexico. Likewise, no significant differences in allele or genotype distributions were observed between melanoma patients and control individuals. These findings suggest that the evaluated *IRF5* genetic variants do not constitute major susceptibility factors for melanoma in this cohort.

Nevertheless, the observed reduction in *IRF5* expression in melanoma samples, together with existing evidence supporting the role of *IRF5* in immune regulation, highlights the relevance of this transcription factor in the immunobiology of melanoma. Overall, the results support a role for *IRF5* as an immune modulator rather than a direct genetic determinant of melanoma risk, underscoring the need for future studies integrating functional, transcriptomic, and immunological approaches to clarify its contribution to melanoma pathogenesis and therapeutic response.

## Figures and Tables

**Table 1:** Sociodemographic characteristics of the study groups.

Characteristic	Melanoma	Reference Group	*p*
*n*	153	221	
Age	62.5 (50–73)	66 (56–72)	0.08
Sex *n* (%)			
Female	90 (59)	133 (60)	0.87
Male	63 (41)	88 (40)	
Skin phototypes			
I	9	0	
II	46	19	
III	41	103	
IV	51	79	
V	4	1	

Clinical characteristics of melanoma patients.

**Table 2:** Clinical characteristics of patients with melanoma.

Characteristic	Level	Female *n* (%)	Male *n* (%)	Total *n* (%)	*p*
Anatomical location	Head/neck	21 (24.41)	24 (40)	45 (30.82)	0.053
	Trunk	14 (16.27)	14 (23.33)	28 (19.17)	
	Upper limbs	19 (22.09)	10 (16.66)	29 (19.86)	
	Lower limbs	32 (37.20)	12 (20)	44 (30.13)	
Histological subtype	Superficial Spreading	33 (41.77)	19 (37.25)	52 (40)	0.574
	Nodular	12 (15.18)	10 (19.60)	22 (16.92)	
	Acral lentiginous	18 (22.78)	8 (15.68)	26 (20)	
	Lentigo maligna	16 (20.25)	14 (27.45)	30 (23.07)	
Breslow thickness	<1 mm	22 (37.28)	12 (28.57)	34 (33.66)	0.543
	1.1–2 mm	9 (15.25)	11 (26.19)	20 (19.80)	
	2.1–4 mm	13 (22.03)	8 (19.04)	21 (20.79)	
	>4 mm	15 (25.42)	11 (26.19)	26 (25.74)	
Clark level	I	4 (8.88)	6 (15.78)	10 (12.04)	0.818
	II	11 (24.44)	11 (28.94)	22 (26.50)	
	III	16 (35.55)	11 (28.94)	27 (32.53)	
	IV	12 (26.66)	8 (21.05)	20 (24.09)	
	V	2 (4.44)	2 (5.26)	4 (4.81)	

**Table 3:** Allelic and genotypic frequencies in the melanoma and reference groups.

	Melanoma n = 150 (%)	Reference Group n = 223 (%)	OR (CI 95%)	*p*
rs3807135 T>C				
Alleles				
T	141(0.47)	183(0.41)	1	-
C	159(0.53)	259(0.58)	1.255 [0.934–1.686]	0.131
Genotypes				
T/T	35(0.23)	34(0.15)	1	-
C/T	71(0.47)	115(0.52)	0.59 [0.34 to 1.04]	0.07
C/C	44(0.29)	72(0.32)	0.59 [0.32 to 1.08]	0.09
HWE				0.56
rs3757385 T>G				
Alleles				
T	141(0.47)	186(0.42)	1	-
G	159(0.53)	256(0.57)	1.22 [0.908–1.639]	0.185
Genotypes				
T/T	35(0.23)	36(0.16)	1	-
T/G	71(0.47)	114(0.51)	0.64 [0.36 to 1.11]	0.11
G/G	44(0.29)	71(0.32)	0.63 [0.35 to 1.15]	0.14
HWE				0.687
rs3778754 C>G				
Alleles				
C	169(0.56)	226(0.51)	1	-
G	131(0.43)	216(0.48)	0.811 [0.604–1.088]	0.163
Genotypes				
C/C	48(0.32)	58(0.262)	1	-
C/G	73(0.486)	110(0.497)	0.80 [0.49 to 1.30]	0.37
G/G	29(0.193)	53(0.239)	0.66 [0.36 to 1.19]	0.17
HWE				0.998

Note. Statistics used: Chi2, Pearson p, odds ratio. Meaning of the abbreviations: HWE: Hardy-Weinberg equilibrium, -: no *p*-value applies because it is the reference allele, OR: Odds Ratio.

**Table 4:** Haplotype frequencies in melanoma and reference groups, and their association with disease status.

Haplotype	Melanoma	Reference Group	OR (CI 95%)	*p*
TTC *	141(0.47)	182(0.41)	1	-
CGG	131(0.43)	216(0.48)	0.78 [0.57–1.06]	0.12
CGC	28(0.09)	39(0.08)	0.92 [0.54–1.57]	0.77

Note. The * highlights the most common haplotype; the hyphen (-) is indicated because no *p*-value applies because it is the reference allele.

**Table 5:** Association between the histopathological subtype of melanoma and Breslow thickness.

Breslow Thickness	Superficial Spreading	Nodular	Acral Lentiginous	Lentigo Malign	*p*
<1 mm	18	1	5	10	<0.01
1.1–2 mm	17	0	2	0	
2.1–4 mm	6	7	5	3	
>4 mm	7	13	5	0	

Note: The table shows the distribution of histopathological melanoma subtypes according to tumor thickness measured by the Breslow index. A statistically significant association was observed between both variables (*p* < 0.01). Nodular melanomas were more frequently observed in higher Breslow thickness categories, whereas superficial spreading melanomas were more frequently observed in lower thickness categories.

**Table 6:** Association between melanoma histopathological subtype and anatomical location.

Anatomical Location	Superficial Spreading	Nodular	Acral Lentiginous	Lentigo Malign	*p*
Head/neck	12	4	0	22	<0.01
Trunk	14	7	0	4	
Upper extremity	12	7	7	1	
Lower extremity	14	5	16	3	

Note: Distribution of melanoma subtypes based on tumor anatomical location. A statistically significant association was identified (*p* < 0.01). Lentigo malign melanoma was more frequently observed on the head and neck, acral lentiginous melanoma on the extremities, and superficial spreading melanoma on the trunk and extremities.

## Data Availability

The original contributions presented in this study are included in the article. Further inquiries can be directed to the corresponding author.
